# Lack of association between type 2 diabetes and major depression: epidemiologic and genetic evidence in a multiethnic population

**DOI:** 10.1038/tp.2015.113

**Published:** 2015-08-11

**Authors:** Z Samaan, S Garasia, H C Gerstein, J C Engert, V Mohan, R Diaz, S S Anand, D Meyre

**Affiliations:** 1 Department of Psychiatry and Behavioral Neurosciences, McMaster University, Hamilton, ON, Canada; 2 Department of Clinical Epidemiology and Biostatistics, Michael DeGroote Centre for Learning and Discovery, McMaster University, Hamilton, ON, Canada; 3 Population Health Research Institute, McMaster University and Hamilton Health Sciences, Hamilton General Hospital, Hamilton, ON, Canada; 4 Department of Medicine, McMaster University, Hamilton, ON, Canada; 5 Department of Medicine, McGill University, Montreal, QC, Canada; 6 Madras Diabetes Research Foundation, Chennai, India; 7 ECLA Academic Research Organization, Rosario, Argentina; 8 Department of Pathology and Molecular Medicine, McMaster University, Hamilton, ON, Canada

## Abstract

The positive association between depression and type 2 diabetes (T2D) has been controversial, and little is known about the molecular determinants linking these disorders. Here we investigated the association between T2D and depression at the clinical and genetic level in a multiethnic cohort. We studied 17 404 individuals from EpiDREAM (3209 depression cases and 14 195 controls) who were at risk for T2D and had both phenotypic and genotypic information available at baseline. The glycemic status was determined using the 2003 American Diabetes Association criteria and an oral glucose tolerance test. Major depressive episode during the previous 12 months was diagnosed using the Diagnostic and Statistical Manual of Mental Disorders, Fourth Edition diagnostic criteria. Twenty single-nucleotide polymorphisms (SNPs) previously associated with T2D were genotyped using the cardiovascular gene-centric 50-K SNP array and were analyzed separately and in combination using an unweighted genotype score (GS). Multivariate logistic regression models adjusted for age, sex, ethnicity and body mass index were performed. Newly diagnosed impaired fasting glucose (IFG)/impaired glucose tolerance (IGT), T2D and dysglycemia status were not associated with major depression (0.30⩽*P*⩽0.65). Twelve out of twenty SNPs and the GS were associated with IFG/IGT, T2D and/or dysglycemia status (6.0 × 10^−35^⩽*P*⩽0.048). In contrast, the 20 SNPs and GS were not associated with depression (*P*⩾0.09). Our cross-sectional data do not support an association between T2D and depression at the clinical and genetic level in a multiethnic population at risk for T2D.

## Introduction

Depression is a chronic disease characterized by the presence of low mood, loss of interests or pleasure, sleep disturbance and appetite changes.^1^ According to the World Health Organization, depression affects approximately 350 million people worldwide, and it is the second leading cause of morbidity globally.^2^ Depression is associated with a 50% increase in the risk of overall mortality, including suicide.^3, 4^ The development of depression is thought to involve an interplay between genetic and environmental factors.^5^ Known risk factors for depression include sex, age, childhood adversity, stressful life events, socioeconomic status, childbirth or medical comorbidities.^6^ Heritability (the fraction of phenotype variability that can be attributed to genetic variation in a population) estimates of 30–40% have been reported for risk of major depressive disorder in family and twin studies.^7^ Thus far, the elucidation of the genetic basis of depression has been surprisingly unsuccessful.^7^ The largest genome-wide association study (GWAS) published to date, which included 9240 major depression cases and 9519 controls in the discovery stage and 6783 major depression cases and 50 695 controls in the replication stage, did not identify genome-wide significant single-nucleotide polymorphisms (SNPs) for depression.^8^

Type 2 diabetes (T2D), the most common type of diabetes, is a metabolic disorder that is characterized by high blood glucose in the context of insulin resistance and relative beta cell dysfunction.^9^ According to the International Federation of Diabetes and the World Health Organization, diabetes affected more than 371 million adults in 2012, with more than 90% of diabetes cases diagnosed as T2D. This number is expected to increase to 552 million adults by 2030, with most of the newly diagnosed cases arising in developing countries. Risk factors for T2D include family history of diabetes, aging, decreasing physical activity and urbanization.^10^ However, the current T2D epidemic is mostly explained by the worldwide rise of obesity in combination with better health care and higher life expectancy of diabetic patients.^11^ T2D is associated with serious health complications and with a 11–14-year loss of life expectancy on average.^12^ The recent explosion of T2D rates has garnered a lot of attention in both the research field and in the media, especially because a lot is still unknown about the pathophysiology of T2D.^9^ Individuals with a family history of T2D are three times more likely to develop the disease than people in the general population, and twin and family-based studies reported heritability estimates for T2D of 30–80%.^13, 14, 15, 16^ Twenty-two loci responsible for monogenic forms of non-autoimmune diabetes (with or without syndrome features) and more than 90 common variants associated with polygenic T2D have been identified to date.^17^

Meta-analyses of longitudinal studies have shown a reciprocal link between T2D and depression.^18, 19, 20^ The association between depression at baseline and risk of incident T2D can result from an unhealthy lifestyle (for example, lack of physical activity and poor diet patterns) and increased obesity risk observed in depressed individuals.^19^ The use of psychotropic medications has also been associated with an increased risk of obesity and T2D.^21, 22^ A low socioeconomic status may indirectly link depression and T2D as it predicts both diseases.^6, 23^ The association between baseline T2D status and increased risk of incident depression can be explained by an increased medical attention brought to T2D patients, or distress associated with T2D diagnosis and its complex self-management.^24, 25, 26^ High chronic levels of stress hormone (for example, cortisol and interleukin-6) have been reported in T2D patients, and have been linked to greater depressive and hostile symptoms and greater stress experience than in healthy controls.^27, 28^ Finally, an alternative explanation of the reciprocal association between T2D and depression may be that both diseases share common molecular roots.^24^ To test this hypothesis, we investigated the effect of 20 T2D predisposing variants separately and together as a gene score on T2D-related traits on the risk of major depression using baseline data of the multiethnic study EpiDREAM.

## Materials and methods

### Participants

EpiDREAM is a longitudinal study that enrolled 24 872 individuals from 21 countries who were at risk for T2D, including subjects who participated in the DREAM clinical trial.^29^ All individuals who were deemed to be at risk for dysglycemia defined by family history, ethnicity and abdominal obesity, between the ages of 18 and 85 years, were screened using a 75-g oral glucose tolerance test (OGTT) from July 2001 to August 2003. Detailed methods and description of the study cohort and inclusion/exclusion criteria have been described earlier.^30, 31^ The current study used cross-sectional data from the baseline screening visit for the EpiDREAM and DREAM studies. We included 17 404 subjects from six ethnic groups (East Asian, South Asian, European, African, Latin American and Native North American) who have both phenotypic and genotypic information available at baseline (Supplementary Figure 1). Self-reported ethnicity has been validated in the 17 404 individuals using the eigenstrat software (http://genepath.med.harvard.edu/~reich/Software.htm). Samples that failed to cluster with individuals of the same self-reported ethnicity were removed. The EpiDREAM and DREAM studies were approved by local ethics committee and informed consent was obtained from each subject before participating in the EpiDREAM or DREAM study, in accordance with the Declaration of Helsinki.

### Genotyping

DNA has been successfully extracted from buffy coats in 19 498 participants of the EpiDREAM study using the Gentra System (Supplementary Figure 1). Genotyping was performed using the Illumina cardiovascular gene-centric bead chip microarray ITMAT Broad Care.^32^ Genotyping was performed at the McGill University and Genome Quebec Innovation Centre using the Illumina Bead Studio genotyping module, version 3.2. We established a list of SNPs that reached genome-wide significance (*P*<5 × 10^−8^) of association with the T2D status in populations of European ancestry. We used three different strategies to optimize the SNP selection procedure using a key word search on (i) the National Human Genome Research Institute GWAS Catalog (www.genome.gov/gwastudies/), (ii) the HuGE Navigator GWAS Integrator (www.hugenavigator.net/HuGENavigator/gWAHitStartPage.do) and (iii) the PubMed database (www.ncbi.nlm.nih.gov/pubmed). Using this strategy, we found 93 independent SNPs associated with T2D in literature. From this list, 20 autosomal SNPs were available on versions 1 and 2 of the ITMAT Broad Care 50-K SNP array (Supplementary Table 1). The 20 SNPs include: rs1260326 (*GCKR*), rs2943634 (*IRS1*), rs1801282 (*PPARγ2*), rs1470579 (*IGF2BP2*), rs1801214 (*WFS1*), rs7754840 (*CDKAL1*), rs1799884 (*GCK*), rs13266634 (*SLC30A8*), rs2383208 (*CDKN2A/B*), rs5015480 (*HHEX*), rs7903146 (*TCF7L2*), rs231362 (*KCNQ1*), rs2283228 (*KCNQ1*), rs5219 (*KCNJ11*), rs10830963 (*MTNR1B*), rs4430796 (*HNF1B*), rs12454712 (*BCL2*), rs16996148 (*GATAD2A*), rs8108269 (*GIPR*) and rs1884614 (*HNF4A*). SNPs obeyed Hardy–Weinberg equilibrium within each ethnic group in the overall sample as well as in the depressed and nondepressed subgroups (*P*⩾0.001, Supplementary Table 1), to the exception of two SNPs. We detected a significant (*P*<0.001) departure from Hardy–Weinberg equilibrium for rs1260326 (*GCKR*) in the Latin American depressed subgroup, and for rs1884614 (*HNF4A*) in the Latin American nondepressed subgroup (Supplementary Table 1). We did not detect significant Hardy–Weinberg disequilibrium for rs1260326 and rs1884614 in the other ethnic groups. Subsequently, the Hardy–Weinberg disequilibrium identified in Latin American populations for these two SNPs did not result from genotyping errors but more likely was the consequence of the admixed nature of this population. As a result, the two SNPs passed the quality-control step and were included in the study. The call rate for each of the 20 SNPs was comprised between 99.33 and 100% (Supplementary Table 1).

### Phenotyping

Demographic data as well as direct anthropometric measurements were obtained from study participants using a standardized protocol. Height (m) and weight (kg) were measured in clinical centers by trained staff. Body mass index (BMI) was calculated as weight in kilograms (kg) divided by height in meter (m) squared. The 2003 American Diabetes Association criteria were used to classify participants as having normal glucose tolerance, impaired fasting glucose (IFG), impaired glucose tolerance (IGT) or T2D at baseline, as confirmed by an OGTT. Normoglycemia was defined as a fasting plasma glucose <5.6 mmol l^−1^, IFG was defined as a fasting plasma glucose of 5.6–6.9 mmol l^−1^, IGT was defined as a fasting plasma glucose below 7.0 mmol l^−1^ and a 2-h glucose between 7.8 and 11.0 mmol l^−1^, and diabetes was defined if either the fasting plasma glucose was ⩾7.0 mmol l^−1^ or the 2-h glucose was ⩾11.1 mmol l^−1^.^33^ Subjects with IFG, IGT or T2D were considered as having dysglycemia.

The assessment of a major depressive episode in the past 12 months was performed at baseline using the Diagnostic and Statistical Manual of Mental Disorders, Fourth Edition diagnostic criteria as described elsewhere.^34^ Interviews were conducted face to face by interviewers trained in the study procedures, and a structured case report form was completed to assess depression (a major depressive episode in the past 12 months). Participants were asked whether they have experienced any of the following symptoms during the past 12 months and whether these symptoms were persistent on a daily basis and lasted for at least 2 weeks: feeling sad, blue or depressed, loss of interest in most things, feeling tired or low energy, changing body weight, having difficulty sleeping, having difficulty concentrating, thinking about death and having low self-esteem or confidence. A depressive episode was considered to be present if the individual had five or more of the above symptoms.

### Statistical analyses

All statistical analyses were performed using the SPSS 14.0 software (IBM, Armonk, NY, USA). The comparison of baseline characteristics between depression cases and controls was made using *t*-tests or *X*^2^-tests. We coded genotypes as 0, 1 and 2, depending on the number of copies of the T2D risk alleles. A genotype score (GS) was calculated by summing the alleles of 20 T2D predisposing SNPs. We used an unweighted genetic score as recommended by Janssens *et al.*^35^ Individuals with more than one missing value were discarded from the calculation of the genetic risk score and the remaining missing values were imputed using the method of the mean. This imputation was performed for each SNP individually in each separate ethnicity using the arithmetic average of the coded genotypes observed for all the successfully genotyped individuals. The association of SNPs/GS with binary traits (for example, T2D or depression) was tested using a logistic regression model. Regression models were adjusted for confounding variables including age, sex, ethnicity, BMI as well as glycemic status (normal glucose tolerance, IFG/IGT and T2D) for specific analyses. Genetic association studies for T2D-related traits were performed under an additive mode of inheritance, as routinely used in GWAS. Genetic association studies for major depression were performed under the additive, dominant and recessive models. Given the high prior likelihood of association between the 20 T2D predisposing SNPs, GS and T2D-related traits, we considered a two-sided *P*<0.05 as significant. The association of T2D-associated SNPs with major depression has never been reported to date and can be considered as a discovery study. Therefore, we used the Bonferroni correction to adjust two-sided *P* for multiple statistical tests. We tested 20 SNPs under additive, dominant and recessive models. It has been shown that testing the three models is equivalent to a correction factor of 2.21.^36^ We also tested the GS under an additive model. A *P*<0.0011 (0.05/45) was considered significant for the genetic association tests with major depression status.

## Results

### Baseline characteristics of major depression cases and controls

Table 1 summarizes the baseline characteristics by major depression status in the EpiDREAM study. From the 17 404 individuals included in the analysis, 3209 were classified as being depressed, whereas 14 195 were not. In the total sample, 60.9% were women and the majority of participants with a major depressive episode were women (75.7%, *P*=1.08 × 10^−93^). The mean age was 53±11 years in EpiDREAM; however, individuals with major depressive disorder were on average 2 years younger than nondepressed controls (*P*=6.92 × 10^−20^). The ethnic distribution was significantly different in the depression and nondepressed groups (*P*=1.98 × 10^−93^). South Asians and East Asians reported less depression, whereas Latin Americans reported more depression. Individuals with major depression displayed on average a slightly lower prevalence of dysglycemia (55.1% versus 57.7%) than nondepressed controls in a univariate analysis (*P*=0.005). A higher BMI was observed in depressed individuals when compared with nondepressed controls (31.58 versus 29.84 kg/m^2^, *P*=4.65 × 10^−40^).

### Association between the glycemic status and major depression

In a multivariate logistic regression model adjusted for sex, age, ethnicity and BMI, IFG/IGT status (*P*=0.50), T2D status (*P*=0.64) and dysglycemia status (*P*=0.48) were not significantly associated with major depressive episode at baseline (Supplementary Table 2). Fasting plasma glucose and 2-h plasma glucose after OGTT were not associated with the depression status (*P*=0.29 and *P*=0.66, respectively, data not shown). The lack of association between T2D and depression observed at the clinical level results from multiple environmental and genetic influences in interplay. It therefore does not imply that individual genetic effects may not be observed in EpiDREAM. The association between 20 T2D-associated SNPs and depression was therefore further investigated.

### Association between T2D predisposing SNPs, GS and glycemic status

Genotype distributions of the 20 T2D predisposing gene variants are presented by the ethnic group in Supplementary Table 1. Associations between the 20 T2D predisposing SNPs, the GS and IFG/IGT, T2D or dysglycemia at baseline adjusted for sex, age, BMI and ethnicity are reported in Table 2. The direction of effect of 19 out of 20 SNPs included in the study was consistent with previous reports, whereby the T2D risk allele increased IFG/IGT, T2D and dysglycemia risks. Twelve out of the twenty SNPs were significantly associated with T2D (*P* comprised between 0.033 and 1.8 × 10^−18^, Table 2). The stronger association was found for rs7903146 SNP in *TCF7L2*. The GS was also significantly associated with increased risk of T2D (odds ratio (OR)=1.11 (1.09–1.13) per additional risk allele, *P*=1.2 × 10^−31^). Eight out of the twenty SNPs were significantly associated with the IFG/IGT status (*P* comprised between 0.039 and 5.7 × 10^−16^, Table 2). Rs10830963 in *MTNR1B* was the more strongly associated SNP with the IFG/IGT status. Four SNPs were significantly associated with T2D but did not achieve significance with the IFG/IGT status (*IRS1* (rs2943634), *CDKAL1* (rs7754840), *CDKN2A/B* (rs2383208) and *KCNJ11* (rs5219), Table 2). The GS was also associated with increased risk of IFG/IGT but the effect size was 57% less compared with that found for T2D (OR=1.07 (1.05–1.08) per additional risk allele, *P*=1.1 × 10^−23^). Ten SNPs associated with T2D also achieved evidence of association with dysglycemia (*P* comprised between 0.048 and 9.3 × 10^−15^, Table 2). The stronger association was found for rs10830963 SNP in *MTNR1B*. The GS was associated with increased risk of dysglycemia (OR=1.07 (1.06–1.09) per additional risk allele, *P*=6.0 × 10^−35^). SNPs in or near *GCKR* (rs1260326), *PPARG* (rs1801282), *WFS1* (rs1801214), *SLC30A8* (rs13266634), *KCNQ1* (rs231362), *BCL2* (rs12454712), *GATAD2A* (rs16996148) or *HNF4A* (rs1884614) were not associated with any T2D-related traits.

### Association between T2D predisposing SNPs and major depression

There was no significant association between the 20 T2D predisposing SNPs and major depression at baseline under the additive, dominant and recessive models after appropriate Bonferroni correction (0.04⩽*P*_unadjusted_⩽0.96; Table 3). The T2D GS was not associated with major depression (OR=1.00 (0.99–1.02) per additional risk allele, *P*=0.83).

## Discussion

Here we investigated the relationships between T2D and major depressive episode in a large cross-sectional multiethnic study. This debated topic is timely and important as these widespread disorders are a growing public health concern at the global level.^18, 19, 20, 24, 25^ Studying 3209 depressed cases and 14 195 nondepressed controls, we were unable to find a significant association between IFG/IGT, T2D and major depressive episode at baseline in the EpiDREAM study. Individuals with major depressive disorder displayed on average a slightly lower prevalence of dysglycemia (55.1% versus 57.7%) than nondepressed controls. However, these associations performed under a univariate model analysis did not remain significant after accounting for confounding factors such as sex, age, BMI and ethnicity in a multivariate regression model. These results are in apparent contradiction with previous meta-analyses of cross-sectional and longitudinal studies that showed a reciprocal positive association between T2D and depression.^18, 19, 20, 37^ However, it is noteworthy that these studies present several limitations. First, the diagnosis of depression status was on the basis of varying methods: diagnosis by a general practitioner (with an unknown method of diagnosis) or self-reported questionnaires.^18, 19, 20^ In the current study, we used a structured interview to ask participants about depressive symptoms lasting at least 2 weeks in the previous 12 months on the basis of the well-recognized Diagnostic and Statistical Manual of Mental Disorders, Fourth Edition criteria. Using this reliable method for diagnosis of depression and considering recent severe episodes of depression lowers the risk of misclassification error and recall bias.^38, 39^ The T2D status in previous reports was on the basis of self-reported information as well, and these studies did not distinguish between newly and previously diagnosed T2D cases.^18, 19, 20^ In contrast, we used the gold standard OGTT method to assess glycemic status and we compared depression prevalence in subjects with normal glucose tolerance, IFG/IGT and T2D, covering a broader range of glycemic status. Owing to the specific characteristics of the EpiDREAM study (participants underwent an OGTT to determine their eligibility in a randomized clinical trial), participants were unaware of their IFG, IGT or T2D status before the study screening and had no T2D treatment.^29^ This prevented us to include depressed cases who have been diagnosed as a result of increased medical attention or psychological distress associated with the onset of T2D and it allowed us to control for recall biases in our study.^40^ Importantly, our data are consistent with a recent meta-analysis by Nouwen *et al.*^25^ showing an absence of association between IFG/IGT, previously undiagnosed T2D subjects and depression. An increased risk of depression in previously diagnosed T2D subjects in comparison with IFG/IGT or previously undiagnosed T2D patients was also demonstrated in this study.^25^ Taking into consideration previous literature and the conclusions of our study, it is tempting to speculate that the link between T2D and incident risk of depression may be the result of an ‘environmental' (detection through increased medical attention/ascertainment biases) cause or residual confounding rather than a shared biological/molecular origin.^20, 24^ An alternative explanation for the lack of consistency between previous reports and our current study may be that the association between T2D and depression described in literature results from a confounding effect. As these previous studies were not adjusted for BMI, and as depression is positively associated with BMI in literature and in our sample, the association between T2D and depression may be indirectly explained by the higher BMI observed in T2D patients.^18, 19, 20, 41^ The EpiDREAM study is enriched in obesity/T2D cases, which prevents the results to be generalized to the general population. Additional studies are needed at this early stage to provide a more definitive answer on this important question.

Our data showing no association between T2D and depression at the clinical level are further supported by the absence of association between 20 T2D predisposing SNPs analyzed individually or together as a T2D GS and major depressive disorder. Previous reports have suggested that the increased prevalence of depression observed in diagnosed T2D may be explained by shared biological/molecular determinants.^24^ Our genetic association data do not support this view. We had a statistical power >80% to detect odds ratio as modest as 1.10 at the nominal level of significance, and 1.15 at the Bonferroni-corrected significant level in a broad range of allele frequencies (Supplementary Figure 2). However, we cannot fully exclude that more subtle genetic effects (OR<1.10) of T2D predisposing SNPs on depression exist and may have not been detected here. Recent simulations have suggested that the SNPs associated with depression have small individual effects and GWAS including >100 000 cases may be needed to identify them.^42^ This study is the first to our knowledge to investigate the association between T2D susceptibility SNPs (analyzed separately and together as a genotype score) and major depression.

Our study has several strengths including a large sample, data from multiple ethnic backgrounds that lead to worldwide relevant conclusions and a well-characterized phenotype. Glycemic status (that is, IFT/IGT and T2D) and major depressive episode were assessed simultaneously at baseline during the study, avoiding recall bias while assessing the association between T2D and depression outcomes. An additional strength of the study relates to the use of a GS to reflect the genetic predisposition to T2D. Lastly, we chose a study enriched by design in patients with IFG/IGT and T2D (42.5% and 14.7%, respectively), which boosts the statistical power for analyzing the association between the glycemic status and depression. As a comparison, a recent report indicated the prevalence of T2D in the multiethnic Canadian population to be 5.6%, 2.6-fold lower than in the EpiDREAM study. Limitations of our study include a nonexhaustive list of T2D-associated SNPs as more than 90 loci have been identified to date in diverse ethnic groups. We acknowledge that we did not investigate the effects of rare variants or mutations contributing to monogenic or polygenic T2D on major depression. Such a project would obviously complement our work. We are aware that testing the association of depression predisposing SNPs on T2D-related traits may have also complemented the present study. However, no genetic variant reaching the genome-wide level of association with depression has been identified to date.^8^ The multiethnic nature of our sample may lower the power to identify ethnic-specific associations, as the relevance of GWAS proxy SNPs identified in European populations may be questioned in other ethnic groups. The fact that the EpiDREAM participants responded on a volunteer basis to advertisements and referrals may have lowered the number of depressive cases who are less likely to participate in such initiatives. Together with the ascertainment of study participants (enrichment in IFG/IGT and T2D cases), this hinders the generalization of our conclusions to a general population. As the diagnosis of depression is restricted to a single episode in the previous year in our study, it is possible that previous episodes of depression may have been missed. EpiDREAM is a longitudinal study; however, the diagnosis of major depressive disorder was assessed at baseline but not after 3.3 years of follow-up. This prevented us to analyze the association between the glycemic status, the 20 T2D-associated SNPs and incident depression, which represents another clear limitation of this study.

To our knowledge, this is the first study that investigated the association between T2D and depression both at the clinical and genetic level in a cross-sectional multiethnic cohort. Our data do not support a shared etiology between T2D and predisposition to major depression.

## Figures and Tables

**Table 1:** Baseline characteristics by depression status in the EpiDREAM study

*Characteristics*	*Categories*	*All*	*Depression case*	*Nondepressed control*	P*-value*^a^
Sex *n* (%)	Female	10 606 (60.9)	2429 (75.7)	8177 (57.6)	1.079 × 10^−93^
	Male	6798 (39.1)	780 (24.3)	6018 (42.4)	
Glycemic status *n* (%)	Normal	7443 (42.8)	1442 (44.9)	6001 (42.3)	0.005
	IFG or IGT	7395 (42.5)	1341 (41.8)	6054 (42.6)	
	Diabetes	2566 (14.7)	426 (13.3)	2140 (15.1)	
Age mean (s.d.)		52.65 (11.37)	51.08 (10.56)	53.01 (11.521)	6.917 × 10^−20^
BMI mean (s.d.)		30.16 (6.22)	31.58 (6.78)	29.84 (6.04)	4.648 × 10^−40^
Ethnicity *n* (%)	South Asian	2760 (15.8)	193 (6.0)	2567 (18.1)	1.980 × 10^−93^
	East Asian	225 (1.3)	23 (0.7)	202 (1.4)	
	European	9378 (53.9)	1750 (54.5)	7628 (53.7)	
	African	1249 (7.2)	227 (7.1)	1022 (7.2)	
	Latin American	3292 (18.9)	914 (28.5)	2378 (16.8)	
	Native North American	500 (2.9)	102 (3.2)	398 (2.8)	
	Total	17 404 (100)	3209 (100)	14 195 (100)	

Abbreviations: BMI, body mass index; IFG, impaired fasting glucose; IGT, impaired glucose tolerance.

a *P*-values are reported for unadjusted univariable analyses (*X*^2^ or Student's *t*-tests).

**Table 2:** Association between SNPs and IFG/IGT, T2D status or dysglycemia status

*Gene*	*SNP*	*Risk allele*	*IFG/IGT status*			*T2D status*			*Dysglycemia status*		
			*OR*	*95% CI*	P*-value*	*OR*	*95% CI*	P*-value*	*OR*	*95% CI*	P*-value*
*GCKR*	rs1260326	C	1.05	1.00–1.10	0.061	0.98	0.91–1.05	0.571	1.04	0.99–1.09	0.102
*IRS1*	rs2943634	C	1.04	0.99–1.10	0.097	**1.13**	**1.05**–**1.22**	**1.4 × 10**^**−3**^	1.05	1.00–1.10	0.052
*PPARG*	rs1801282	C	1.04	0.97–1.13	0.271	1.11	0.99–1.24	0.070	1.06	0.99–1.14	0.090
*IGF2BP2*	rs1470579	C	**1.08**	**1.03**–**1.13**	**0.002**	**1.22**	**1.14**–**1.30**	**7.4 × 10**^**−9**^	**1.11**	**1.07**–**1.17**	**2.5 × 10**^**−6**^
*WFS1*	rs1801214	T	1.01	0.96–1.06	0.718	1.06	0.98–1.13	0.140	1.02	0.97–1.07	0.451
*CDKAL1*	rs7754840	C	1.05	1.00–1.10	0.068	**1.15**	**1.07**–**1.24**	**1.2 × 10**^**−4**^	**1.08**	**1.03**–**1.13**	**1.1 × 10**^**−3**^
*GCK*	rs1799884	A	**1.21**	**1.13**–**1.28**	**3.7 × 10**^**−9**^	**1.16**	**1.07**–**1.27**	**6.5 × 10**^**−4**^	**1.20**	**1.14**–**1.28**	**5.7 × 10**^**−10**^
*SLC30A8*	rs13266634	C	1.03	0.98–1.09	0.242	1.03	0.96–1.12	0.411	1.04	0.98–1.09	0.171
*CDKN2A/B*	rs2383208	A	1.03	0.97–1.10	0.312	**1.17**	**1.07**–**1.28**	**8.6 × 10**^**−4**^	1.06	1.00–1.12	0.051
*HHEX*	rs5015480	C	**1.06**	**1.01**–**1.11**	**0.014**	**1.10**	**1.03**–**1.18**	**5.7 × 10**^**−3**^	**1.08**	**1.03**–**1.13**	**1.1 × 10**^**−3**^
*TCF7L2*	rs7903146	T	**1.15**	**1.09**–**1.21**	**2.4 × 10**^**−7**^	**1.39**	**1.29**–**1.49**	**1.3 × 10**^**−18**^	**1.21**	**1.15**–**1.27**	**7.4 × 10**^**−14**^
*KCNQ1*	rs231362	C	1.00	0.95–1.05	0.954	1.04	0.97–1.12	0.220	1.01	0.97–1.06	0.653
*KCNQ1*	rs2283228	A	**1.18**	**1.09**–**1.28**	**3.1 × 10**^**−5**^	**1.14**	**1.02**–**1.27**	**0.024**	**1.14**	**1.06**–**1.23**	**3.7 × 10**^**-4**^
*KCNJ11*	rs5219	T	1.04	0.99–1.09	0.149	**1.10**	**1.03**–**1.18**	**0.008**	**1.05**	**1.00**–**1.10**	**0.048**
*MTNR1B*	rs10830963	G	**1.24**	**1.18**–**1.31**	**5.7 × 10**^**−16**^	**1.19**	**1.10**–**1.28**	**5.6 × 10**^**−6**^	**1.23**	**1.17**–**1.29**	**9.3 × 10**^**−15**^
*HNF1B*	rs4430796	G	**1.05**	**1.01**–**1.10**	**0.039**	**1.10**	**1.03**–**1.18**	**0.007**	**1.07**	**1.03**–**1.12**	**0.002**
*BCL2*	rs12454712	T	1.03	0.98–1.08	0.279	1.03	0.96–1.10	0.401	1.04	0.99–1.08	0.113
*GATAD2A*	rs16996148	T	1.07	0.99–1.17	0.098	1.00	0.89–1.13	0.960	1.06	0.98–1.14	0.164
*GIPR*	rs8108269	G	**1.06**	**1.01**–**1.11**	**0.037**	**1.08**	**1.01**–**1.16**	**0.033**	**1.06**	**1.01**–**1.11**	**0.011**
*HNF4A*	rs1884614	T	0.97	0.92–1.03	0.362	1.01	0.94–1.10	0.705	0.98	0.93–1.03	0.468
Gene score			**1.07**	**1.05**–**1.08**	**1.1 × 10**^**−23**^	**1.11**	**1.09**–**1.13**	**1.2 ×** ^**10-31**^	**1.07**	**1.06**–**1.09**	**6.0 × 10**^**−35**^

Abbreviations: BMI, body mass index; CI, confidence interval; IFG, impaired fasting glucose; IGT, impaired glucose tolerance; OR, odds ratio; SNP, single-nucleotide polymorphism; T2D, type 2 diabetes.

All *P*-values adjusted for sex, age, ethnicity and BMI. Bold values indicate significance at *P*<0.05.

**Table 3:** Association between T2D predisposing SNPs, genotype score and major depressive disorder under the additive, dominant and recessive models

*Gene*	*SNP*		*Additive*			*Dominant*			*Recessive*		
		*Risk allele*	*Odds ratio*	*95% Confidence interval*	P*-value*	*Odds ratio*	*95% Confidence interval*	P*-value*	*Odds ratio*	*95% Confidence interval*	P*-value*
*GCKR*	rs1260326	C	0.951	0.897–1.008	0.090	0.912	0.816–1.019	0.105	0.950	0.874–1.032	0.226
*IRS1*	rs2943634	C	1.036	0.975–1.100	0.258	0.991	0.872–1.125	0.888	1.068	0.985–1.158	0.108
*PPARγ2*	rs1801282	C	1.029	0.940–1.127	0.539	0.828	0.590–1.164	0.277	1.051	0.951–1.161	0.330
*IGF2BP2*	rs1470579	C	1.039	0.979–1.102	0.211	1.040	0.958–1.129	0.351	1.071	0.952–1.205	0.254
*WFS1*	rs1801214	T	1.011	0.953–1.072	0.714	0.972	0.864–1.094	0.642	1.034	0.954–1.121	0.412
*CDKAL1*	rs7754840	C	1.002	0.945–1.063	0.943	1.011	0.933–1.096	0.785	0.983	0.870–1.112	0.790
*GCK*	rs1799884	A	1.008	0.939–1.082	0.822	0.996	0.916–1.084	0.934	1.092	0.892–1.337	0.392
*SLC30A8*	rs13266634	C	0.975	0.915–1.039	0.429	0.909	0.783–1.054	0.207	0.986	0.910–1.069	0.741
*CDKN2A/B*	rs2383208	A	0.997	0.927–1.073	0.937	1.075	0.858–1.346	0.531	0.986	0.905–1.073	0.738
*HHEX*	rs5015480	C	0.992	0.938–1.050	0.792	1.073	0.970–1.186	0.170	0.933	0.856–1.017	0.113
*TCF7L2*	rs7903146	T	0.989	0.931–1.051	0.722	0.997	0.921–1.079	0.940	0.954	0.832–1.095	0.503
*KCNQI*	Rs231362	C	1.029	0.972–1.090	0.325	0.959	0.864–1.065	0.437	1.092	1.005–1.188	0.039
*KCNQI*	rs2283228	A	0.950	0.870–1.036	0.245	0.760	0.579–0.998	0.048	0.968	0.876–1.070	0.527
*KCNJ11*	rs5219	T	0.987	0.930–1.047	0.671	0.979	0.901–1.063	0.607	0.993	0.882–1.118	0.912
*MTNR1B*	rs10830963	G	1.003	0.942–1.068	0.932	1.002	0.924–1.087	0.964	1.009	0.874–1.164	0.908
*HNF1B*	rs4430796	G	0.967	0.914–1.023	0.246	0.978	0.895–1.069	0.623	0.932	0.846–1.026	0.151
*BCL2*	Rs12454712	T	1.013	0.956–1.074	0.656	0.976	0.873–1.090	0.664	1.040	0.959–1.129	0.342
*GATAD2A*	rs16996148	T	0.953	0.862–1.054	0.350	0.958	0.859–1.069	0.444	0.812	0.528–1.250	0.344
*GIPR*	rs8108269	G	1.033	0.974–1.096	0.281	1.014	0.936–1.098	0.735	1.113	0.986–1.256	0.083
*HNF4A*	rs1884614	T	1.042	0.977–1.112	0.211	1.054	0.971–1.145	0.211	1.050	0.901–1.224	0.531
Gene score			1.002	0.987–1.016	0.833	—	—	—	—	—	—

Abbreviations: BMI, body mass index; SNP, single-nucleotide polymorphism; T2D, type 2 diabetes.

All *P*-values adjusted for sex, age, ethnicity and BMI.
