# Supplementary material for: Lack of association between type 2 diabetes and major depression: epidemiologic and genetic evidence in a multiethnic population
Source: Transl Psychiatry. 2015 Aug 11;5(8):e618–. doi: 10.1038/tp.2015.113 (PMC4564566; doi:10.1038/tp.2015.113)

Supplementary Table 1. Genotype distributions of 20 T2D predisposing SNPs in the EpiDREAM Study.

				All						Depression cases						Depression controls					
Ethnicity	Major allele	Minor allele	T2D risk allele	Genotype count			T2D risk allele frequency	Call rate (%)	HWE *P*-value	Genotype count			T2D risk allele frequency	Call rate (%)	HWE *P*-value	Genotype count			T2D risk allele frequency	Call rate (%)	HWE *P*-value
Rs1260326 –***GCKR***																					
				CC	CT	TT				CC	CT	TT				CC	CT	TT			
South Asian	C	T	C	1715	929	115	0.790	99.96	0.441	129	60	4	0.824	100	0.324	1586	869	111	0.787	99.96	0.558
East Asian				73	108	44	0.564	100	0.721	10	8	5	0.609	100	0.196	63	100	39	0.559	100	0.952
European				3134	4581	1661	0.579	99.98	0.854	555	877	318	0.568	100	0.379	2579	3704	1343	0.581	99.97	0.835
African				957	271	21	0.875	100	0.720	168	53	6	0.857	100	0.466	789	218	15	0.879	100	0.989
Latin American				1292	1485	514	0.618	99.97	0.011	378	381	155	0.622	100	$6.0x10^{-4}$	914	1104	359	0.617	99.96	0.393
Native				237	211	52	0.685	100	0.621	43	49	10	0.662	100	0.460	194	162	42	0.691	100	0.349
Total				7408	7585	2407	0.644	99.98	N.A.	1283	1428	498	0.622	100	N.A.	6125	6157	1909	0.648	99.97	N.A.
Rs2943634 – *IRS1*																					
				CC	CT	TT				CC	CT	TT				CC	CT	TT			
South Asian	C	T	C	1737	901	117	0.794	99.82	0.991	126	58	9	0.803	100	0.490	1611	843	108	0.793	99.81	0.863
East Asian				186	38	1	0.911	100	0.522	19	4	0	0.913	100	0.648	167	34	1	0.911	100	0.601
European				4169	4148	1048	0.667	99.86	0.737	784	755	208	0.665	99.83	0.206	3385	3393	840	0.667	99.87	0.814
African				236	611	400	0.434	99.84	0.921	34	124	68	0.425	99.56	0.065	202	487	332	0.436	99.90	0.333
Latin American				1764	1246	273	0.727	99.73	0.012	517	321	70	0.746	99.34	0.044	1247	925	203	0.720	99.87	0.093
Native				320	154	26	0.794	100	0.191	74	23	5	0.838	100	0.089	246	131	21	0.783	100	0.517
Total				8412	7098	1865	0.688	99.83	N.A.	1554	1285	360	0.687	99.69	N.A.	6858	5813	1505	0.689	99.87	N.A.
Rs1801282 – *PPARG*																					
				CC	CG	GG				CC	CG	GG				CC	CG	GG			
South Asian	C	G	C	2146	570	44	0.089	100	0.386	148	42	3	0.876	100	0.992	1998	528	41	0.881	**100**	0.369
East Asian				203	22	0	0.951	100	0.441	21	2	0	0.956	100	0.827	182	20	0	0.950	100	0.459
European				7283	1969	126	0.882	100	0.586	1381	344	25	0.887	100	0.499	5902	1625	101	0.880	100	0.362
African				1206	43	0	0.983	100	0.536	221	6	0	0.987	100	0.840	985	37	0	0.982	100	0.556
Latin American				2637	611	43	0.894	99.97	0.262	726	172	16	0.888	100	0.123	1911	439	27	0.896	99.96	0.751
Native				400	95	5	0.895	100	0.807	80	20	2	0.882	100	0.575	320	75	3	0.898	100	0.539
Total				13875	3310	218	0.892	99.99	N.A.	2577	586	46	0.894	100	N.A.	11298	2724	172	0.657	99.99	N.A.
Rs1470579 – *IGF2BP2*																					
				AA	AC	CC				AA	AC	CC				AA	AC	CC			
South Asian	A	C	C	765	1312	676	0.484	99.75	0.016	52	93	48	0.490	100	0.618	713	1219	628	0.483	99.73	0.018
East Asian				119	89	17	0.273	100	0.949	9	12	2	0.348	100	0.472	110	77	15	0.265	100	0.764
European				4279	4094	1004	0.325	99.99	0.595	788	762	200	0.332	100	0.444	3491	3332	804	0.324	99.99	0.831
African				72	432	744	0.769	99.92	0.377	7	75	145	0.804	100	0.468	65	357	599	0.761	99.90	0.233
Latin American				1519	1437	333	0.320	99.91	0.800	417	407	89	0.320	99.89	0.474	1102	1030	244	0.319	99.92	0.884

Native				304	171	25	0.221	100	0.880	59	38	5	0.235	100	0.722	245	133	20	0.217	100	0.724
Total				7058	7535	2799	0.378	99.93	N.A.	1332	1387	489	0.369	99.97	N.A.	5726	6148	2310	0.380	99.92	N.A.
Rs1801214 – *WFS1*																					
				TT	CT	CC				TT	CT	CC				TT	CT	CC			
South Asian	T	C	T	1547	1021	191	0.746	99.96	0.204	118	66	9	0.782	100	0.953	1429	955	182	0.743	99.96	0.196
East Asian				189	32	4	0.911	100	0.067	20	2	1	0.913	100	0.030	169	30	3	0.911	100	0.226
European				3470	4526	1380	0.611	99.98	0.123	656	822	270	0.610	99.89	0.637	2814	3704	1110	0.612	100	0.053
African				606	520	123	0.693	100	0.460	117	84	26	0.700	100	0.075	489	436	97	0.692	100	0.990
Latin American				1485	1432	374	0.669	99.97	0.307	422	397	95	0.679	100	0.909	1063	1035	279	0.665	99.96	0.265
Native				223	203	74	0.649	100	0.015	42	42	18	0.618	100	0.195	181	161	56	0.657	100	0.041
Total				7520	7734	2146	0.654	99.98	N.A.	1375	1413	419	0.649	99.94	N.A.	6145	6321	1727	0.656	99.99	N.A.
Rs7754840 – *CDKAL1*																					
				GG	GC	CC				GG	GC	CC				GG	GC	CC			
South Asian	G	C	C	1584	1000	175	0.245	99.96	0.310	111	66	16	0.254	100	0.176	1473	934	159	0.244	99.96	0.501
East Asian				99	92	34	0.356	100	0.106	13	10	0	0.217	100	0.183	86	82	34	0.371	100	0.064
European				4232	4092	1054	0.331	100	0.172	783	790	177	0.327	100	0.279	3449	3302	877	0.331	100	0.043
African				229	612	408	0.572	100	0.985	44	108	75	0.568	100	0.647	185	504	333	0.572	100	0.812
Latin American				1557	1380	355	0.317	100	0.061	422	382	110	0.329	100	0.103	1135	998	245	0.313	100	0.244
Native				206	237	57	0.351	100	0.366	48	43	11	0.667	100	0.77	158	194	46	0.359	100	0.241
Total				7907	7413	2083	0.333	99.99	N.A.	1421	1399	389	0.339	100	N.A.	6486	6014	1694	0.331	99.99	N.A.
Rs1799884 – *GCK*																					
				GG	GA	AA				GG	GA	AA				GG	GA	AA			
South Asian	G	A	A	2113	597	49	0.126	99.96	0.366	152	36	5	0.119	100	0.121	1961	561	44	0.126	99.96	0.596
East Asian				157	62	6	0.164	100	0.967	16	6	1	0.174	100	0.659	141	56	5	0.163	100	0.840
European				6317	2748	302	0.179	99.88	0.881	1171	524	53	0.180	99.89	0.542	5146	2224	249	0.179	99.88	0.646
African				830	364	55	0.190	100	0.065	155	59	13	0.187	100	0.028	675	305	42	0.190	100	0.312
Latin American				1977	1129	183	0.227	99.91	0.193	551	302	59	0.230	99.78	0.047	1426	827	124	0.226	99.96	0.773
Native				328	158	14	0.186	100	0.330	63	37	2	0.2010	100	0.191	265	121	12	0.182	100	0.685
Total				11722	5058	609	0.180	99.91	N.A.	2108	964	133	0.192	99.88	N.A.	9614	4094	476	0.178	99.92	N.A.
Rs13266634 – *SLC30A8*																					
				CC	CT	TT				CC	CT	TT				CC	CT	TT			
South Asian	C	T	C	1700	929	131	0.484	100	0.777	117	67	9	0.780	100	0.880	1583	862	122	0.785	100	0.738
East Asian				75	108	41	0.576	99.56	0.846	10	10	3	0.652	100	0.842	65	98	38	0.567	99.50	0.921
European				4719	3847	812	0.708	100	0.482	877	711	162	0.704	100	0.303	3842	3136	650	0.709	100	0.778
African				1045	189	15	0.912	100	0.056	190	32	5	0.907	100	0.016	855	157	10	0.913	100	0.356
Latin American				1889	1182	221	0.753	100	0.052	521	328	65	0.749	100	0.179	1368	854	156	0.755	100	0.148
Native				273	191	36	0.737	100	0.744	52	42	8	0.716	100	0.905	221	149	28	0.742	100	0.674
Total				9701	6446	1256	0.743	99.99	N.A.	1767	1190	252	0.736	100	N.A.	7934	5256	1004	0.744	99.99	N.A.
Rs2383208– *CDKN2A/B*																					
				AA	AG	GG				AA	AG	GG				AA	AG	GG			
South	A	G	A	2006	690	62	0.852	99.93	0.769	145	42	6	0.860	100	0.184	1861	648	56	0.852	99.92	0.963

Asian																					
East Asian				119	81	25	0.709	100	0.055	12	10	1	0.739	100	0.541	107	71	24	0.705	100	0.028
European				6474	2587	314	0.828	99.97	0.005	1194	499	56	0.825	99.94	0.662	5280	2088	258	0.829	99.97	0.004
African				831	373	45	0.815	100	0.695	159	61	7	0.835	100	0.698	672	312	38	0.810	100	0.811
Latin American				2252	919	120	0.824	99.97	0.031	621	263	30	0.823	100	0.739	1631	656	90	0.824	99.96	0.020
Native				367	125	8	0.859	100	0.474	77	25	0	0.877	100	0.158	290	100	8	0.854	100	0.856
Total				12049	4775	574	0.830	99.97	N.A.	2208	900	100	0.829	99.97	N.A.	9841	3875	474	0.830	99.96	N.A.
Rs5015480– *HHEX*																					
				CC	CT	TT				CC	CT	TT				CC	CT	TT			
South Asian	C	T	C	446	1361	953	0.408	100	0.277	33	107	53	0.448	100	0.093	413	1254	900	0.405	100	0.494
East Asian				10	67	148	0.193	100	0.497	2	8	13	0.261	100	0.638	8	59	135	0.186	100	0.629
European				3325	4504	1549	0.595	100	0.718	609	857	284	0.593	100	0.546	2716	3647	1265	0.595	100	0.491
African				470	601	178	0.617	100	0.524	79	117	31	0.606	100	0.233	391	484	147	0.619	100	0.888
Latin American				953	1585	754	0.530	100	0.054	232	474	208	0.513	100	0.252	721	1111	546	0.537	100	0.003
Native				109	226	165	0.440	100	0.059	21	43	38	0.417	100	0.051	88	183	127	0.451	100	0.154
Total				5313	8344	3747	0.545	100	N.A.	976	1606	627	0. 554	100	N.A.	4337	6738	3120	0.543	100	N.A.
Rs7903146 – *TCF7L2*																					
				CC	CT	TT				CC	CT	TT				CC	CT	TT			
South Asian	C	T	T	1314	1174	272	0.311	100	0.680	86	86	21	0.332	100	0.942	1228	1088	251	0.310	100	0.658
East Asian				201	22	2	0.058	100	0.126	18	4	1	0.130	100	0.263	183	18	1	0.050	100	0.450
European				4518	3904	956	0.310	100	0.009	849	731	170	0.306	100	0.490	3669	3173	786	0.311	100	0.010
African				615	522	112	0.299	100	0.935	112	93	22	0.302	100	0.675	503	429	90	0.298	100	0.914
Latin American				1705	1307	280	0.284	100	0.190	483	359	72	0.275	100	0.643	1222	948	208	0.287	100	0.214
Native				345	145	10	0.165	100	0.241	68	32	2	0.176	100	0.423	277	113	8	0.162	100	0.365
Total				8698	7074	1632	0.297	100	N.A.	1616	1305	288	0.293	100	N.A.	7082	5769	1344	0.298	100	N.A.
Rs231362– *KCNQ1*																					
				CC	CT	TT				CC	CT	TT				CC	CT	TT			
South Asian	C	T	C	1562	1017	178	0.751	99.89	0.472	101	80	12	0.740	100	0.462	1461	937	166	0.752	99.88	0.341
East Asian				187	37	1	0.913	100	0.561	20	3	0	0.935	100	0.738	167	34	1	0.911	100	0.601
European				2662	4657	2055	0.532	99.96	0.830	507	840	403	0.530	100	0.126	2155	3817	1652	0.533	99.95	0.619
African				770	412	67	0.781	100	0.225	150	67	10	0.808	100	0.476	620	345	57	0.775	100	0.326
Latin American				1281	1498	507	0.618	99.82	0.047	398	384	130	0.647	99.78	0.018	883	1114	377	0.607	100	0.412
Native				203	228	69	0.6340	100	0.697	46	43	13	0.662	100	0.556	157	185	56	0.627	100	0.899
Total				6665	7849	2877	0.609	99.93	N.A.	1222	1417	568	0.602	99.94	N.A.	5443	6432	2309	0.610	99.92	N.A.
Rs2283228– *KCNQ1*																					
				AA	AC	CC				AA	AC	CC				AA	AC	CC			
South Asian	A	C	A	2626	130	4	0.975	100	0.076	186	6	1	0.979	100	0.001	2440	124	3	0.975	100	0.279
East Asian				91	110	24	0.649	100	0.274	12	9	2	0.717	100	0.867	79	101	22	0.641	100	0.219
European				8056	1285	37	0.927	100	0.060	1502	239	9	0.927	100	0.878	6554	1046	28	0.928	100	0.044
African				978	258	13	0.886	100	0.377	183	42	2	0.898	100	0.810	795	216	11	0.884	100	0.385

Latin American				2169	968	155	0.806	100	0.001	577	278	59	0.783	100	0.002	1592	690	96	0.815	100	0.054
Native				281	172	47	0.734	100	0.008	51	42	9	0.706	100	0.933	230	130	38	0.741	100	0.003
Total				14201	2923	280	0.900	100	N.A.	2511	616	82	0.878	100	N.A.	11690	2307	198	0.905	100	N.A.
Rs5219 – *KCNJ11*																					
				CC	CT	TT				CC	CT	TT				CC	CT	TT			
South Asian	C	T	T	1160	1200	385	0.359	99.46	0.009	77	87	28	0.372	99.48	0.671	1083	1113	357	0.358	99.45	0.009
East Asian				101	90	33	0.348	99.56	0.086	9	10	3	0.364	95.65	0.933	92	80	30	0.346	100	0.074
European				3621	4425	1320	0.377	99.87	0.588	695	829	226	0.366	100	0.386	2926	3596	1094	0.380	99.84	0.839
African				1100	141	7	0.062	99.92	0.288	199	28	0	0.062	100	0.322	901	113	7	0.062	99.90	0.102
Latin American				1376	1465	445	0.358	99.82	0.080	379	387	146	0.372	99.78	0.005	997	1078	299	0.353	99.96	0.774
Native				216	215	69	0.353	100	0.190	46	46	10	0.323	100	0.760	170	169	59	0.361	100	0.114
Total				7574	7536	2259	0.347	99.80	N.A.	1405	1387	413	0.345	99.88	N.A.	6169	6149	1846	0.347	99.78	N.A.
Rs10830963 – *MTNR1B*																					
				CC	CG	GG				CC	CG	GG				CC	CG	GG			
South Asian	C	G	G	934	1324	502	0.422	100	0.386	56	88	49	0.518	100	0.227	878	1236	453	0.417	100	0.617
East Asian				76	108	41	0.422	100	0.808	8	15	0	0.326	100	0.020	68	93	41	0.433	100	0.375
European				4534	3958	886	0.305	100	0.601	857	731	162	0.301	100	0.734	3677	3227	724	0.306	100	0.678
African				1087	152	10	0.069	100	0.072	201	26	0	0.057	100	0.360	886	126	10	0.071	100	0.024
Latin American				1899	1207	186	0.240	100	0.749	534	335	45	0.232	100	0.414	1365	872	141	0.243	100	0.911
Native				296	168	36	0.240	100	0.078	57	38	7	0.255	100	0.846	239	130	29	0.236	100	0.059
Total				8826	6917	1661	0.294	100	N.A.	1713	1233	263	0.274	100	N.A.	7113	5684	1398	0.299	100	N.A.
Rs4430796 – *HNF1B*																					
				AA	AG	GG				AA	AG	GG				AA	AG	GG			
South Asian	A	G	G	1327	1143	278	0.309	99.56	0.169	103	71	18	0.279	99.48	0.267	1224	1072	260	0.311	99.57	0.264
East Asian				107	96	22	0.311	100	0.945	13	7	3	0.283	100	0.232	94	89	19	0.314	100	0.754
European				2446	4667	2213	0.487	99.45	0.886	438	887	417	0.494	99.54	0.440	2008	3780	1796	0.486	99.42	0.835
African				148	544	548	0.661	99.28	0.467	30	109	85	0.623	98.68	0.594	118	435	463	0.670	99.41	0.306
Latin American				1029	1573	677	0.446	99.61	0.093	302	445	163	0.424	99.56	0.967	727	1128	514	0.455	99.62	0.052
Native				174	246	80	0.406	100	0.654	39	47	16	0.388	100	0.769	135	199	64	0.411	100	0.512
Total				5231	8269	3818	0.459	99.51	N.A.	925	1566	702	0.465	99.50	N.A.	4306	6703	3116	0.458	99.51	N.A.
Rs12454712 – *BCL2*																					
				TT	TC	CC				TT	TC	CC				TT	TC	CC			
South Asian	T	C	T	524	1313	923	0.428	100	0.138	38	89	66	0.427	100	0.421	486	1224	857	0.428	100	0.188
East Asian				57	109	59	0.496	100	0.642	5	11	7	0.456	100	0.862	52	98	52	0.500	100	0.673
European				3630	4352	1396	0.619	100	0.121	695	791	264	0.623	100	0.115	2935	3561	1132	0.618	100	0.333
African				758	429	62	0.779	100	0.897	136	78	13	0.771	100	0.683	622	351	49	0.780	100	0.954
Latin American				1401	1478	413	0.650	100	0.450	379	417	118	0.643	100	0.844	1022	1061	295	0.653	100	0.445
Native				170	236	94	0.576	100	0.451	47	40	15	0.657	100	0.189	123	196	79	0.555	100	0.954
Total				6540	7917	2947	0.603	100	N.A.	1300	1426	483	0.627	100	N.A.	5240	6491	2464	0.598	100	N.A.
Rs16996148– *GATAD2A*																					

				GG	GT	TT				GG	GT	TT				GG	GT	TT			
South Asian	G	T	T	2108	595	54	0.127	99.89	0.116	140	50	2	0.141	99.48	0.283	1968	545	52	0.126	99.92	0.050
East Asian				190	31	4	0.087	100	0.052	22	1	0	0.022	100	0.915	168	30	4	0.094	100	0.068
European				7962	1348	65	0.079	99.97	0.337	1501	241	8	0.073	100	0.614	6461	1107	57	0.080	99.96	0.207
African				896	312	40	0.157	99.92	0.049	165	53	9	0.1564	100	0.083	731	259	31	0.157	99.90	0.173
Latin American				2854	414	22	0.070	99.92	0.103	800	107	7	0.066	100	0.109	2054	307	15	0.071	99.92	0.342
Native				451	48	1	0.050	99.94	0.814	93	9	0	0.044	100	0.641	358	39	1	0.051	100	0.954
Total				14461	2748	186	0.090	99.95	N.A.	2721	461	26	0.080	99.97	N.A.	11740	2287	160	0.092	99.94	N.A.
Rs8108269 – *GIPR*																					
				TT	TG	GG				TT	GT	GG				TT	GT	GG			
South Asian	T	G	G	1187	1223	320	0.341	98.91	0.854	93	74	24	0.319	98.96	0.133	1094	1149	296	0.343	98.91	0.829
East Asian				80	95	48	0.428	99.11	0.052	5	10	8	0.565	100	0.580	75	85	40	0.413	99.01	0.082
European				4470	3963	893	0.308	99.45	0.734	832	739	170	0.690	99.49	0.752	3638	3224	723	0.308	99.44	0.822
African				444	583	216	0.408	99.52	0.302	87	98	42	0.401	100	0.127	357	485	174	0.410	99.41	0.672
Latin American				1359	1494	417	0.356	99.33	0.839	359	416	132	0.375	99.23	0.519	1000	1078	285	0.349	99.37	0.832
Native				133	258	105	0.472	99.20	0.331	23	54	24	0.505	99.02	0.485	110	204	81	0.463	99.25	0.444
Total				7673	7616	1999	0.336	99.33	N.A.	1399	1391	400	0.343	99.41	N.A.	6274	6225	1599	0.334	99.32	N.A.
Rs1884614–*HNF4A*																					
				CC	CT	TT				CC	CT	TT				CC	CT	TT			
South Asian	C	T	T	1235	1217	308	0.332	100	0.753	87	81	25	0.339	100	0.374	1148	1136	283	0.331	100	0.938
East Asian				64	126	35	0.436	100	0.037	7	12	4	0.435	100	0.768	57	114	31	0.436	100	0.036
European				6261	2817	299	0.182	99.99	0.408	1137	547	66	0.194	100	0.983	5124	2270	233	0.179	99.99	0.336
African				1010	222	17	0.102	100	0.232	178	45	4	0.117	100	0.560	832	177	13	0.099	100	0.307
Latin American				1446	1390	456	0.350	100	4.0×10^{-5}	392	394	128	0.356	100	0.073	1054	996	328	0.347	100	2.0×10^{-4}
Native				174	213	111	0.437	99.60	0.004	36	43	23	0.441	100	0.149	138	170	88	0.437	99.50	0.011
Total				10190	5985	1226	0.242	99.98	N.A.	1837	1122	250	0.253	100	N.A.	8353	4863	976	0.240	99.98	N.A.

Supplementary Table 2. Association between traits related to T2D and major depressive disorder status.

Status	Odds Ratio	95% Confidence Interval	*P*-value
IFG/IGT	0.97	0.89-1.06	0.505
T2D	0.97	0.85-1.10	0.645
Dysglycemia	0.97	0.89-1.05	0.483

Supplementary Figure 1. EpiDREAM study selection process overview.

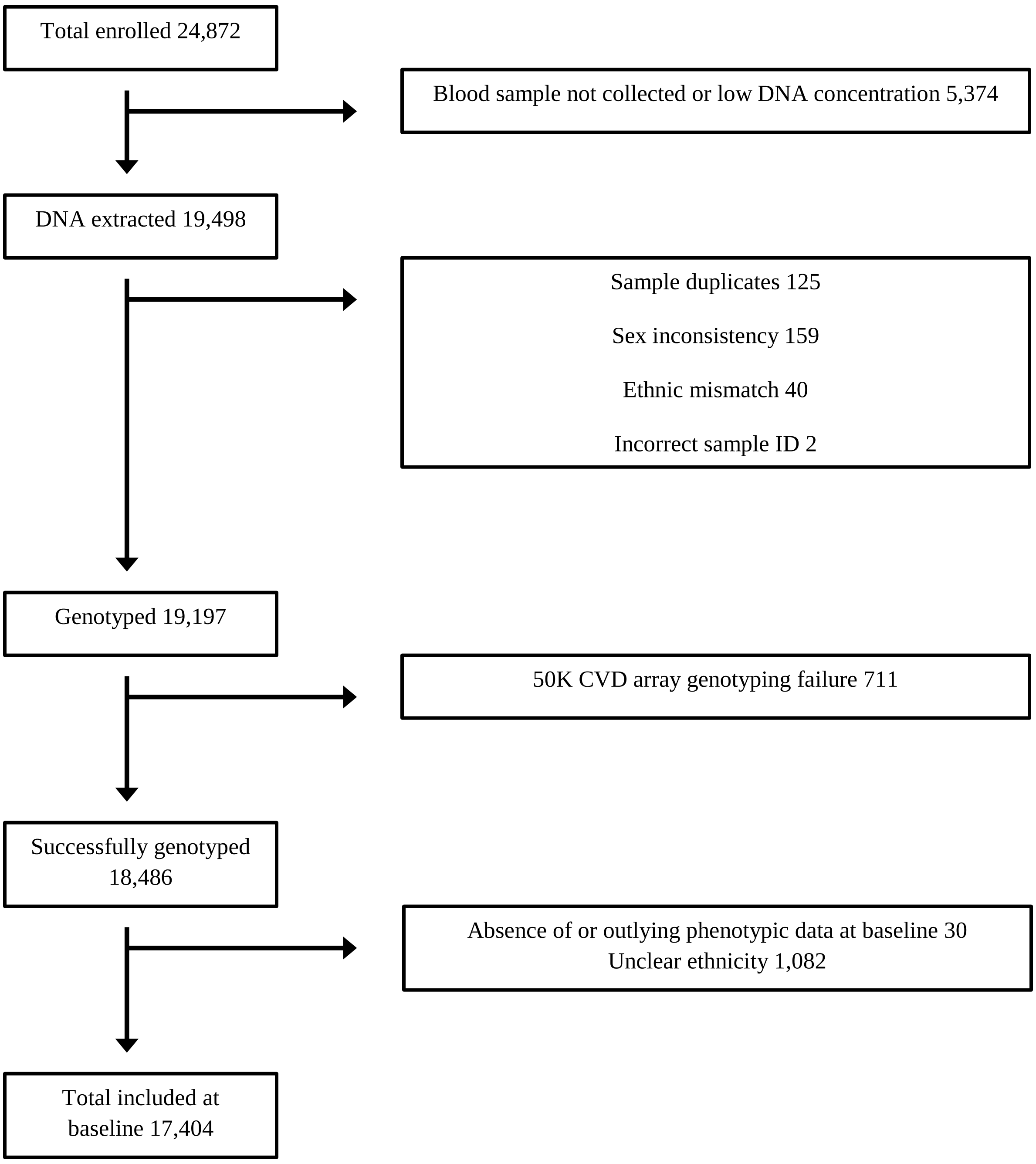

Supplementary Figure 2. Case numbers needed to achieve 80% of statistical power according to allele frequency and odds-ratio. The horizontal line corresponds to the EpiDREAM study (3 209 depression cases and 14 195 non-depressed controls). **A**-Number of cases needed for a statistical power of 80% according to risk allele frequency and odds ratio, assuming a two-sided *P*-value of 0.05 unadjusted for multiple testing and an unequal number of cases and controls (1 *versus* 4.4). **B-**Number of cases needed for a statistical power of 80% according to risk allele frequency and odds ratio, assuming a two-sided P-value of 0.05 after adjustment for multiple testing (corresponding to an unadjusted P-value of 0.0011) and an unequal number of cases and controls (1:4.4).

A

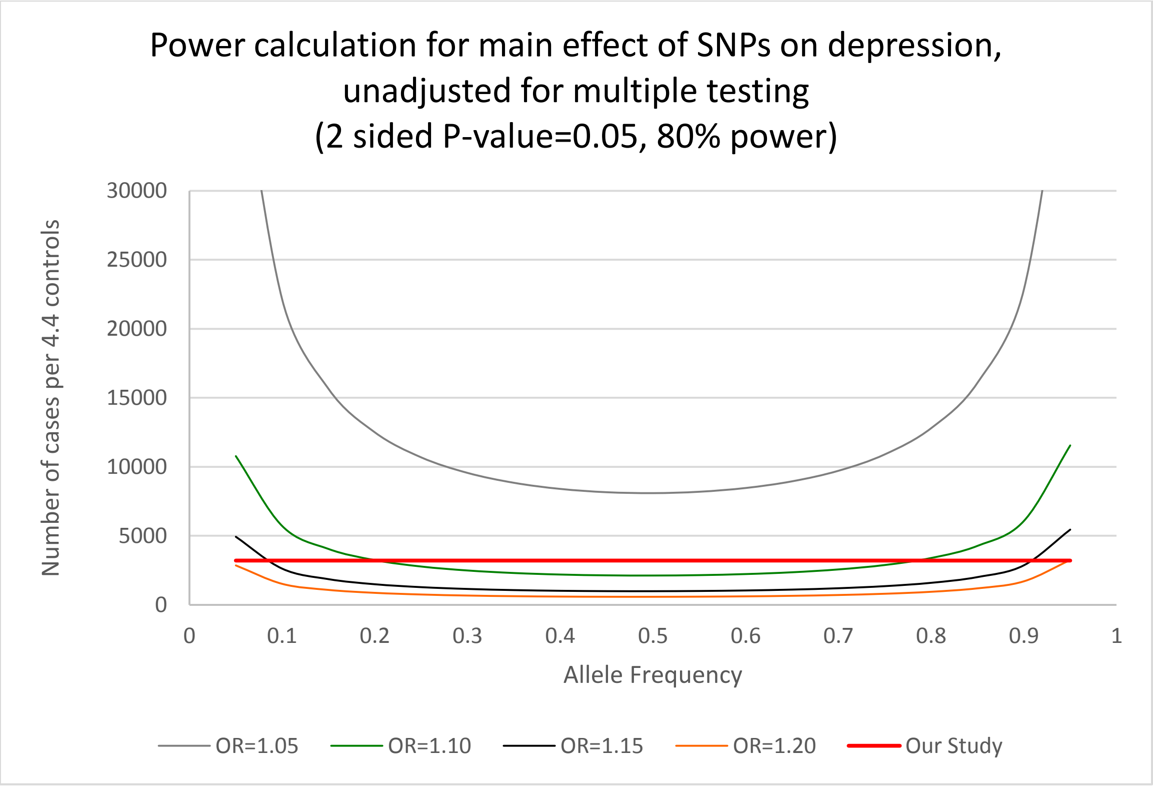

B

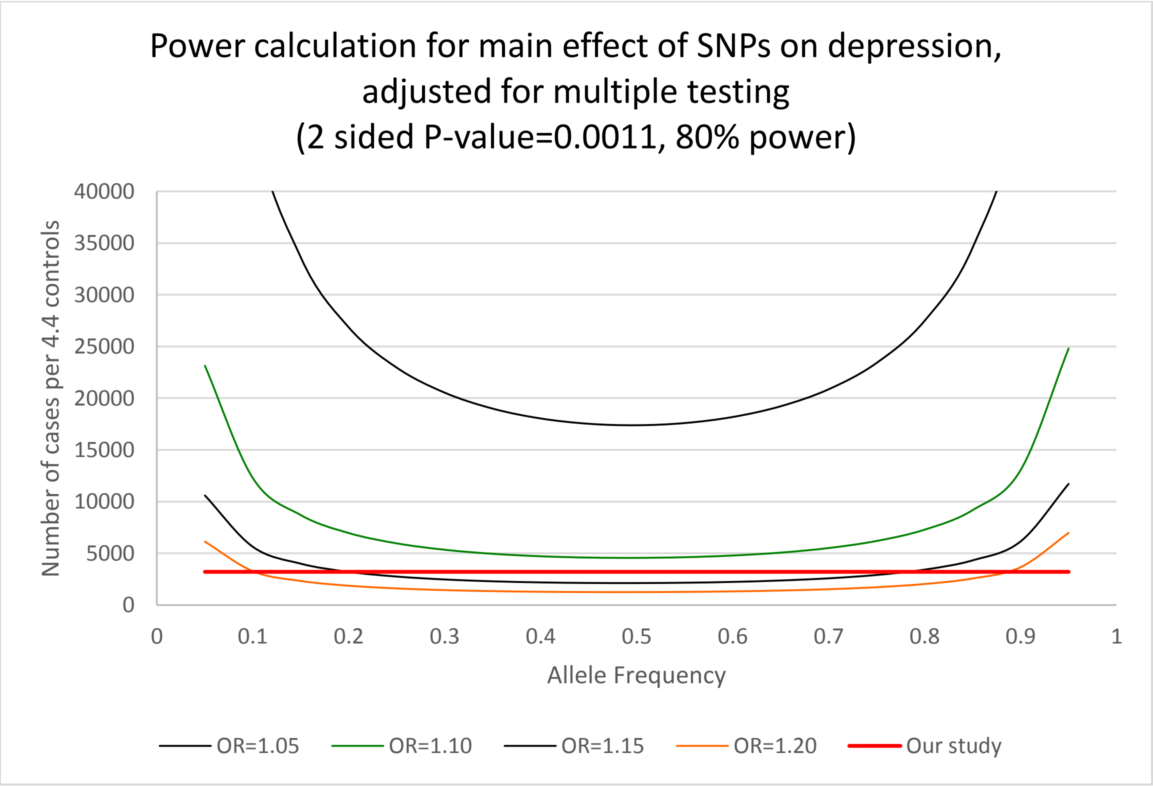

Supplement: Supplementary Information [file tp2015113x1.pdf]
